# Predictors of mortality among HIV infected children on anti-retroviral therapy in Mekelle Hospital, Northern Ethiopia: a retrospective cohort study

**DOI:** 10.1186/1471-2458-13-1047

**Published:** 2013-11-06

**Authors:** Aregay Gebremedhin, Solomon Gebremariam, Fisaha Haile, Berhe Weldearegawi, Carla Decotelli

**Affiliations:** 1 Tigray Regional Health Bureau, Mekelle, Ethiopia; 2 College of Health Sciences Department of Public Health, Mekelle University, Mekelle, Ethiopia; 3 General Manager of Corporate Health at Vale, Vale, Brazil

**Keywords:** Children, HIV, HAART, Survival, Ethiopia

## Abstract

**Background:**

The introduction of antiretroviral therapy in 1996 improved the longevity and wellbeing of peoples living with HIV in the industrialized world including children. This survival benefit of antiretroviral therapy (ART) in reducing HIV related deaths has been well studied in the developed world. In resource-poor settings, where such treatment was started recently, there is inadequate information about impact of ART on the survival of patients especially in children. So, this study aims to investigate predictors of mortality of children on ART. Therefore, the objective of this study was to identify predictors of mortality among children on HAART.

**Methods:**

A retrospective cohort study was conducted on 432 children who initiated antiretroviral therapy from June 2006 to June 2011 at pediatrics ART clinic in Mekelle Hospital, Northern-Ethiopia. Data were extracted from electronic and paper based medical records database and analyzed using Kaplan Meier survival and Cox proportional hazard model to identify independent predictors of children’s mortality on ART.

**Results:**

The total time contributed by the study participants were 14,235 child-months with median follow up of 36 months. The mortality rate of this cohort was 1.40 deaths per 1000 child-months or 16.85 deaths per 1000 child-years. Age less than 18 months [ Adj.HR (95% CI) = (4.39(1.15-17.41)], CD4 percentage <10 [Adj.HR (95% CI) = 2.98(1.12-7.94)], WHO clinical stage (III&IV) [Adj.HR (95% CI) = 4.457(1.01-19.66)], chronic diarrhea[Adj.HR (95% CI) = 4.637(1.50-14.31)] and hemoglobin < 8 g/dl[Adj.HR (95% CI) = 3.77(1.29-10.98)] all at baseline were significantly and independently associated with survival of children on ART.

**Conclusions:**

Mortality of children on ART was low and factors that affect mortality of children on ART were age less than 18 months, lower CD4 percentage, advanced WHO clinical stage (III&IV), presence of chronic diarrhea and lower hemoglobin level all at baseline. The high early mortality rate would support the value of an earlier treatment start before development of signs of immunodeficiency syndrome despite the method of HIV diagnosis and WHO stage.

## Background

The introduction of antiretroviral viral therapy (ART) in 1996 has improved survival and quality of HIV-infected patients in the developed countries. This survival benefit of highly active antiretroviral therapy (HAART) in reducing HIV related deaths has been well studied in the developed world. In resource-poor settings however, where such treatment was started recently, knowledge on treatment results is limited. Moreover, mortality has been high in the first month of ART initiation. But factors contributing to high mortality are poorly investigated [1-3]. About 1,400 children under the age of 15 are infected with HIV every day and approximately 90% of these infections occur in sub-Saharan Africa. Without appropriate care and treatment, more than 50% of newly infected children die before celebrating their second birthday [4-6].

The use of ART has dramatically reduced HIV-associated mortality and morbidity among children in resource-rich settings. Pediatric HIV has been transformed from rapidly fatal infection of early childhood to a manageable chronic disease. For this reason, many HIV infected children are aging into adolescence and early adulthood [3-5]. But according to American Academy of Pediatrics, there might be differences from country to country in many ways such as nutritional status, racial or ethnic and gender differences in total lymphocyte count and CD4+ cell count [6].

Findings from studies in Africa and other low income countries also showed that there was good result of the ART programs i.e. the mortality of children on ART has decreased [7-10]. But in sub-Saharan Africa including Ethiopia, there is substandard information about treatment results and the impact of ART on mortality of children. The independent predictors of survival among children living with HIV and on ART remain poorly characterized in Ethiopia. Therefore, the available evidence in Ethiopia regarding the determinants of survival among children less than 15 years old is inadequate. For this reason, this study dares to investigate the predictors of survival among under 15 year old children on ART.

## Methods

### Study area and period

The study was conducted in Mekelle Hospital 783kms far north of Addis Ababa. Mekelle Hospital serves as a referral hospital for Tigray region and neighboring regions (Afar and Amhara) with a catchment population of 8 million. ART program was introduced in 2003 as fee service and the free ART program started in March 2005. A total of 660 HIV infected children were enrolled and 510 of them were started on ART since ART started in Mekelle Hospital (June 2006-June 2011). Diagnosis of HIV in children was done based on antibody test at 18 months of age. Records of children who start treatment between June 1, 2006 and may 31, 2011 were retrieved.

### Study design and sampling

A retrospective cohort study was conducted among 432 records of under 15 years old children on ART. The patients’ ART identification numbers were used to extract the necessary information from the different ART recording formats. Socio-demographic characteristics, baseline clinical and laboratory measurement information, and treatment outcomes were abstracted from patients’ cards and database and the primary outcome measure was patient mortality (Time death). Seventy eight Transferred in children on ART were excluded because it was impossible to get their baseline information.

### Data collection and quality control

A standard checklist was used for recording information extracted from database and patient cards. This form is developed using the standardized ART entry and follow up form employed by the ART clinic. The laboratory results of CD4 count recorded before starting ART were used as a baseline values. If there is no pre-treatment laboratory test, however, results obtained within one month of ART initiation were considered as baseline values. Four experienced ART nurses who were trained on comprehensive HIV care and involved in patient follow ups collected the data and data collection was supervised by two trained supervisors. During the data collection process the filled checklist was checked for their completeness, consistency and accuracy by the principal investigator every day. Mode, median and mean values was used to address incompleteness, inconsistencies and inaccuracy depending on the measurement scale. The data were entered and cleaned using SPSS version 16.0 by data clerks and exported to STATA version 11.0 for analysis.

### Statistical analysis

Descriptive statistics such as median, mean, standard deviation and proportions were used to describe the general characteristics of the cohort. Person-months/years of follow up were calculated by assessing the date of enrollment for ART and death or censoring. The role of the variables on patient survival was analyzed using Kaplan-Meier survival analysis method. Log Rank test was used to test the equality of survival probabilities and compared across the different groups of covariates. The overall survival function and separate estimates for the stratum of covariates were considered as statistically significant at p-value < 0.05 in the Log- rank test. Hazard ratios (HR) with 95% confidence intervals were used as effect measures. Multivariable Cox proportional hazards regression was used to assess the effect of baseline predictors on the survival of children on ART. Variables with p <0.05 in bivariate analysis were taken to multivariate analysis to estimate hazard ratios with 95% confidence intervals for the mortality rate among children on ART for the covariates at their ART initiation. Model adequacy was assessed using Schoenfeld residuals [11].

### Ethical considerations

Ethical clearance was obtained from the Institutional review board (IRB) of Mekelle University. All information collected from patients cards were kept strictly confidential and names of children or their parents were not included in the abstracted data.

## Results

### Base line cohort characteristics

A total of 432 study participants (children under 15 years old) were included in the study and 416 (96.3%) of the 432 records were included in the analysis. The sample comprised 216 (53%) females and 200 (47%) males. The ages of the cohort at ART initiation were relatively late with median age of 53 months with IQR (24–96) months. Clinically 405 (97.40%) of the children were started on first line ART regimen while the rest started on second line. During the ART initiation 54 (12.9%) of children had tuberculosis out of these 2 were died. Additionally 236 (56.7%) had chronic diarrhea and 4 were died during the follow up time (Table 1).

**Table 1:** Base line socio-demographic and clinical characteristics of children on ART, Mekelle, hospital, and Northern Ethiopia

**Baseline variables**	**Survival status, N = 416**					
	**Censored**		**DEAD**		**Total**	
	**N**	**%**	**N**	**%**	**N**	
**Sex (N = 416)**						
Male	190	48.00	10	50.00	200	48.10
Female	206	52.00	10	50.00	216	51.90
**Age at ART initiation (N = 416)**						
≥120 months	56	14.14	3	15.00	59	14.18
60-120 months	140	35.35	4	20.00	144	34.62
36-59 months	69	17.42	1	5.00	70	16.83
18-35 months	73	18.43	4	20.00	77	18.51
<18 months	58	14.65	8	40.00	66	15.87
**Primary care giver (N = 402)**						
Parents	294	77.00	18	90.0	312	77.60
Relatives	66	17.30	1	5.00	67	16.70
Guardians & Orphan	22	5.80	1	5.00	23	5.70
Missing					14	3.36
**TB at baseline (416)**						
Yes	112	28.30	15	75.00	127	30.53
No	284	71.72	5	25.00	289	69.47
**Baseline WHO clinical stage (N = 416)**						
stage I & II	93	23.50	1	5.0	94	22.60
stage III	187	47.20	14	70.0	201	48.30
stage IV	116	29.30	5	25.0	121	29.10
**CD4 percentage (N = 412)**						
≥10%	223	56.89	6	30	229	55.58
<10%	169	43.11	14	70	183	44.42
**Hemoglobin level (N = 408)**						
≥8 g/dl	363	93.56	15	75	378	92.65
<8 g/dl	25	6.44	5	25	30	7.35

### Survival pattern of the cohort

**Figure 1:**
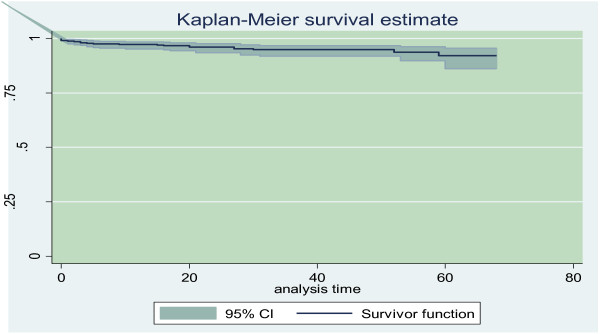
Overall Kaplan - Meier probability of survival curve with 95% confidence intervals of children on ART, Mekelle Hospital, and Northern Ethiopia.

Out of the 416 cohort of children on ART, 313 (75.24%) were alive, 20 (4.81%) had died, 23 (5.53%) lost to follow up, and 60 (14.42%) were transferred out. After initiation of ART, children were followed for a median survival of 36 months with IQR of 17 to 50 months and the cohort contributed to a total of 14,235 person-months of follow up. Mortality rate in this cohort was found to be 1.40 per 1000 child-months. Out of the 20 children who died, 11 [2.39 per 1000 child-months (95% CI, 1.33, 4.32)] died within 12 months of ART initiation while 9 [ 0.93 per 1000 child-months (95% CI, 0.48, 1.79) ] died after 12 months (Figure 1).

### Predictors of mortality

In bivariate analysis, factors associated with mortality were: Hgl level <= 8 g/dl, presence of chronic diarrheal disease, CD4 percentage <10% at ART initiation, WHO clinical stage (III&IV) and age less 18 months. Those patients who have Hgl level <=8 g/dl were four times more likely to die than Hgl level > 8 g/dl at baseline (crude hazard ratio(CHR)) [CHR (95% CI) = 3.77 (1.29,10.98)]. Patients who have chronic diarrheal disease during treatment initiation were five times more likely to die compared with those who have not [CHR (95% CI) = 4.64 (1.27,10.06)]. Those patients whose CD4 cell percent was <= 10% was three times more likely to die than those patients whose CD4 percent was greater than 10% [CHR (95% CI) = 2.98 (1.12,7.94)]. Moreover, those patients with WHO stage (III and IV) were nine times more likely to die than patients on stage (I and II) [CHR (95% CI) = 8.87(1.09,71.96) ].But In multivariate analysis five baseline characteristics, Age < 18 months, WHO clinical stage III and IV, presence of chronic diarrhea, CD4 <= 10 percent and hemoglobin <8 gm/dl were found to be the independent predictors of mortality (Table 2).

**Table 2:** Predictors of mortality among a sample of HIV infected cohorts of children on anti-retroviral treatment in Mekelle hospital, Northern Ethiopia

**Variables**	**P-value**	**HR**		**[95% CI]**
**Age in months at ART initiation**				
≥120		1.00		
60-120	0.365	0.499	0.111	2.248
36-59	0.305	0.303	0.031	2.963
18-35	0.941	0.943	0.201	4.433
<18	0.036	4.387	1.150	17.410
**Gender**		1.00		
Male	0.518	0.723	0.271	1.932
Female				
**TB at baseline**				
No		1.00		
Yes	0.864	1.143	0.248	5.276
**Primary care giver**				
Parents		1.00		
Relatives	0.132	0.209	0.027	1.605
Guardian/orphan	0.343	0.329	0.033	3.271
**Chronic Diarrhea**				
No	1.00	1.00		
Yes	0.008	4.637	1.502	14.314
**Hemoglobin level**				
≥8 g/dl				
<8 g/dl	0.015	3.769	1.294	10.979
**CD4 percentage**				
≥10%		1.00		
<10%	0.029	2.976	1.115	7.944
**WHO clinical stage**				
Stage I & II		1.00		
Stage III & IV	0.048	4.457	1.010	19.664

## Discussion

The findings of this study indicated that from the registered cohort, there were 20 deaths in 1186.25 person-years of retrospective follow up, providing an incidence density of 16.85 deaths per 1000 child-years of death. About 23(5.53%) patients were lost to follow up. Mortality was higher in the first 12 months after ART initiation (2.39 death per 1000 child months) But the over all mortality was 1.40 deaths per 1000 child-months. The over all mortality rate was actually lower when compared with two different studies done in Kenya which reported an overall mortality of the cohort of children on ART as 47 and 84 deaths per 1000 child years [12,13].

Early mortality (death <18 months after ART initiation) was higher than late mortality (death >= 18 months after ART initiation) in this study. This finding is consistent with a study done in USA and ten European countries and South Africa which showed that most of the deaths occurred in the first 6 months following ART initiation [14-16]. Children in WHO clinical stage IV and with severe immunodeficiency had a significant risk of early death at 3 or 6 months [17-19]. This indicated that early HIV diagnosis and early antiretroviral therapy might not be well adopted as recommend which could have reduced early infant mortality by 76% in this study [20,21].

Mortality rate among <= 18 months old children on ART was 56.80 deaths per 1000 child years which was higher as compared with the age 120 months or above i.e. 16.06 deaths per 1000 child-years. This study was in line with a study done in Zambia that showed, as age increases mortality rate will decreased [22]. Moreover, a study done in Malawi ,South Africa and Democratic Republic Congo also revealed that younger age (<18 months) and late WHO stage were more likely to die [23-25]. However, this finding contrasts with studies conducted in Uganda and Zambia that showed children initiated ART at an older age were associated with higher mortality [26,27].

Children with hemoglobin level < 8 g/l were four times more likely to die than those with ≥ 8 g/dl. When results of this study was compared against a 4 years clinical cohort study in Kenya and Zambia among <15 years aged children, children with baseline hemoglobin level of ≤ 8 g/l was two times more likely to die than children with hemoglobin level >8 gm/dl [13,22].

Children who initiated ART at WHO clinical stage (III/IV) were associated with higher mortality. Studies done in South Africa pointed out that, advanced disease (WHO stage III&IV) had an increased risk of mortality [23,28]. Moreover, studies from Cote d’Ivoire, Malawi, and Ethiopia stated that children with advanced HIV disease and very low CD4% have been found to be at highest risk of early mortality following HAART initiation [18,29,30].

Hemoglobin level ≤ 8 gm/dl, CD4 count percent ≤50/ul was found to be significant predictors. This is consistent with studies conducted in Addis Ababa, Ethiopia at Zewditu memorial hospital and All African Leprosy and Rehabilitation Centre (ALERT hospital), showed that severe wasting, severe immunodeficiency (absolute CD4) and low hemoglobin value and, lower age and immunosuppression (low CD4 count) were associated with mortality [31,32].

The sensitivity analysis was performed including the composite endpoint of lost to follow-up (LTFU) and death. The proportion of the lost to follow-up included as being dead were 10.34% (3.02 per 1000 child-months) almost doubled the mortality rate. Age less than 18 months, hemoglobin level, CD4 < 10%, were significantly associated with death. Similar study conducted in South Africa displayed that age less than 18 months, CD4 < 10% ,weight for age Z-score, and female gender were significantly associated with mortality [16]. A study from ten treatment programs in 4 countries (South Africa, Malawi, Mozambique, and Zimbabwe) showed that ignoring LTFU led to substantial underestimation of mortality [33]. Finally data on conrimoxazol prophylaxis was not collected in this study because it was started 3 years after the start date of this study. But a study done among Zambian children on ART reported that Daily cotrimoxazole prophylaxis has been shown to substantially reduce non–Pneumocystis jiroveci–related deaths and hospital admissions in children after infancy, and it is a recommended standard of care for all HIV-infected children. For this reason, this study recommends a further study on added impact of cotrimoxazole prophylaxis among children who are receiving ART [34].

The main weakness of the study is the composition of the study participants with almost half of the children being 5 years or more. As the mortality of HIV infected children is higher in lower age groups this may underestimate the estimates because many younger patients with poor prognosis were probably not included as they died early or were not linked to the program. Finally retrospective cohort study design also limits our ability to gather data about factors that may influence the risk of mortality, for example factors such as lack of family supports, cares given at family level etc.

## Conclusion

In conclusion, findings of this study indicated that mortality in this cohort was found to be 4.8% deaths or 1.40 per 1000 child-months on ART. Finally, age less than 18 months, WHO clinical stage III & IV, chronic diarrhea and hemoglobin < 8 g/dl at baseline remained independent predictors of mortality. The high early mortality in the present study would support the value of an earlier treatment initiation before development of signs of immunodeficiency. Generally, it is sensible to give due emphasis about comprehensive early HIV treatment, care and support for all children on ART to improve survival of children on ART. Finally further study is needed about the effect of cotrimoxazol prophylaxis on mortality of children on ART.

## Competing interests

The authors declare that they have no competing interests.

## Authors’ contributions

AG: Involved in proposal writing, designing, and recruitment and training of Supervisors and data collectors, analysis and write-up and in all stages of the project implementation. He did most of the analysis and write up of the paper. FH contributed in the designing of the methodology, lead investigator and involved in designing of project proposal, design of questionnaires, supervision and involved in the analysis stage of the project and preparation and final approval of the manuscript. SG: contributed in the designing of the methodology, lead investigator and involved in designing of project proposal, design of questionnaires, supervision and involved in the analysis stage of the project. BW: Read and approved the manuscript. CD: Involved in analysis stage of the project. Finally all authors have been read and approved the manuscript.

## Pre-publication history

The pre-publication history for this paper can be accessed here:

http://www.biomedcentral.com/1471-2458/13/1047/prepub
